# Modeling Gene Networks in* Saccharomyces cerevisiae* Based on Gene Expression Profiles

**DOI:** 10.1155/2015/621264

**Published:** 2015-12-14

**Authors:** Yulin Zhang, Kebo Lv, Shudong Wang, Jionglong Su, Dazhi Meng

**Affiliations:** ^1^College of Mathematics and Systems Science, Shandong University of Science and Technology, Qingdao, Shandong 266590, China; ^2^School of Mathematical Sciences, Ocean University of China, Qingdao 266100, China; ^3^College of Computer and Communication Engineering, China University of Petroleum, Qingdao, Shandong 266580, China; ^4^Department of Mathematical Sciences, Xi'an Jiaotong-Liverpool University, Suzhou, Jiangsu 215123, China

## Abstract

Detailed and innovative analysis of gene regulatory network structures may reveal novel insights to biological mechanisms. Here we study how gene regulatory network in* Saccharomyces cerevisiae* can differ under aerobic and anaerobic conditions. To achieve this, we discretized the gene expression profiles and calculated the self-entropy of down- and upregulation of gene expression as well as joint entropy. Based on these quantities the uncertainty coefficient was calculated for each gene triplet, following which, separate gene logic networks were constructed for the aerobic and anaerobic conditions. Four structural parameters such as average degree, average clustering coefficient, average shortest path, and average betweenness were used to compare the structure of the corresponding aerobic and anaerobic logic networks. Five genes were identified to be putative key components of the two energy metabolisms. Furthermore, community analysis using the Newman fast algorithm revealed two significant communities for the aerobic but only one for the anaerobic network. David Gene Functional Classification suggests that, under aerobic conditions, one such community reflects the cell cycle and cell replication, while the other one is linked to the mitochondrial respiratory chain function.

## 1. Introduction

The difference between aerobic and anaerobic conditions at the molecular level has drawn considerable attention in last twenty years. Man and Pilpel [1] found that the transcription rate of mitochondrial gene under aerobic conditions is significantly higher than that under anaerobic conditions, while glycolytic genes are more active under anaerobic conditions. Hou et al. [2] found that under anaerobic conditions SPT3 and SPT15 are overexpressed, which may not only enhance resistance to ethanol and stress but also upregulate the fermentation transcription factors in* Saccharomyces cerevisiae*. Jiang et al. [3] found that, under anaerobic condition, mitochondrial function is weakened while fermentation capacity is enhanced in* Saccharomyces cerevisiae*. This suggests competition between aerobic and anaerobic metabolisms as a result of evolution.

A fundamental goal of genomics is to understand how gene regulatory networks actually give rise to cellular phenotypes. Modeling and reconstruction of gene regulatory networks based on high-throughput data have become one of the most common goals of systems biology. Diverse models of gene regulatory networks have been developed such as Boolean network [4–6], probabilistic Boolean network (PBN) [7, 8], and Bayesian network [9–12]. The main objective of these network models is to study the logical interactions of genes based on large-scale microarray data and further get meaningful biological information. Recently using network method to study gene interactions of* Saccharomyces cerevisiae* has attracted the interest of several researchers [13–16]. For example, Zhang et al. [14] examined the integrated gene interaction network of* Saccharomyces cerevisiae* and found many enriched multicolor network motifs corresponding to different biological themes. They concluded that significantly enriched motifs in the network are often signatures of network themes, higher-order network structures that correspond to biological phenomena. Lee et al. [15] modified a probabilistic functional gene network of the baker's yeast,* Saccharomyces cerevisiae*, and experimentally verified the function of the yeast RNA binding protein Puf6 in 60S ribosomal subunit biogenesis. Hu et al. [16] profiled transcriptional responses in* Saccharomyces cerevisiae* strains with individual deletions of 263 transcription factors. Then they reconstructed a functional transcriptional regulatory network between these transcription factors and analyzed the enrichment of promoter motifs on these transcription factors.

In 2005, Bowers et al. [17] introduced a computational approach called logic analysis of phylogenetic profiles (LAPP), which identified detailed logic relationships among gene triplets on the basis of genomic data. This method may be used for functional annotation of proteins and genes and for designing biochemical experiments to elucidate biological mechanisms. Lately, further progress has been achieved on the theory and application of higher logic. Zhang et al. [18] described a three-way gene interaction model that captures the dynamics of coexpression relationships between two genes. Shoemaker and Panchenko [19] proposed ways to address the defects of the LAPP method, such as high computational complexity, strong dependence on information spectrum, and the uncertainty of homology detection at large genetic distances. Sprinzak et al. [20] detected coordinated regulation of multiple protein complexes using logic analysis of gene expression data and identified protein complexes by mapping specific kinds of gene triples to multicomplexes triplets. Notably, the LAPP method is related to stochastic logic. Modeling approaches using stochastic logic, such as stochastic Boolean network (SBN) and stochastic multiple-valued network (SMN), have been already proposed [21–24].

In this paper, we focus on the construction, analysis, and comparisons of structural characteristics of logic networks inferred from the gene expression profiles of* Saccharomyces cerevisiae* under aerobic and anaerobic conditions. Firstly, gene expression profiles are discretized into multiple values. Secondly, the logical AND, OR, and NOT operators are given by algebraic formulation on multiple values. Down- and upregulation self-entropy as well as joint entropy are used to compute the uncertainty coefficient. Four parameters of the logic network are generalized to more complex networks for our purpose. Putative regulator genes of respiratory mechanisms are identified by contrasting the differences of the four structural parameters. Lastly, the Newman fast algorithm is used to discover the community structures of the logic network. We find that the gene logic network under aerobic condition (aerobic network) has two significant communities while the anaerobic network only has one in this framework. Furthermore, David Gene Functional Classification (http://david.abcc.ncifcrf.gov) [25] reveals the possible biological function of these communities.

## 2. Materials and Methods

### 2.1. Expression Data

The gene expression profiles data of DNA chip in this study are taken from the GSE11452 database using GPL90 platform in the National Center for Biotechnology Information (http://www.ncbi.nlm.nih.gov) (NCBI) and comprise 42 and 52 expression vectors with more than 20,000 genes in yeast under aerobic and anaerobic conditions (Table 1). Such a large number of genes incur an intractable computational complexity, so we select candidate genes based on the Wilcoxon rank sum test [26] as follows. Let *X*_*j*_ = (*x*_*j*1_, *x*_*j*2_,…, *x*_*jn*_0__),  *Y*_*j*_ = (*y*_*j*1_, *y*_*j*2_,…, *y*_*jn*_1__) denote *n*_0_ and *n*_1_-sized random sample of expression of gene *j* under aerobic and anaerobic conditions, respectively. The Wilcoxon rank sum test statistic is derived from the concatenation of *X*_*j*_ and *Y*_*j*_, which results in vector *Z*_*j*_. This is done by first sorting all points in *Z*_*j*_ by ascending order, obtaining the sets of ranks for the *X*_*j*_ and *Y*_*j*_ points, respectively, then calculating the corresponding sums *T*_*j*0_ and *T*_*j*1_, and finally defining the test statistic as *T*_*j*0_/*n*_0_ − *T*_*j*1_/*n*_1_. Under the null hypothesis the expected value of this statistic is zero; otherwise the alternative hypothesis holds, in which case *H*_0_: *T*_*j*0_/*n*_0_ − *T*_*j*1_/*n*_1_ = 0; *H*_1_: *T*_*j*0_/*n*_0_ − *T*_*j*1_/*n*_1_ ≠ 0; then gene *j* is called a candidate gene. Setting the significance level to be 10^−5^, we obtain 73 candidate genes finally.

### 2.2. Methods

#### 2.2.1. Construction of the Gene Logic Network

The expression profile of candidate gene *m* containing *n* samples is denoted in this work by the vector (*d*_*m*1_,…, *d*_*mp*_,…, *d*_*mn*_). To discretize this vector, we define *x*_*i*_ = (*i* − 1)/2*k*, *x* ^*i*^ = 1 − (*i* − 1)/2*k*, *i* = 1,2,…, *k*,  *k* ∈ *N* ^+^, where the tuning parameter *k* controls granularity on the interval [0, 1]. Then for (*d*_*m*1_,…, *d*_*mp*_,…, *d*_*mn*_), we set *d*_*mp*_′ = *x*_*i*_ if *d*_*mp*_ ∈ [(*i* − 1)/2*k*, *i*/2*k*], (1 ≤ *p* ≤ *n*), and similarly *d*_*mp*_′ = *x* ^*i*^ if *d*_*mp*_ ∈ [1 − *i*/2*k*, 1 − (*i* − 1)/2*k*], obtaining the discretized expression vector *D* = (*d*_*m*1_′,…, *d*_*mp*_′,…, *d*_*mn*_′). In order to calculate first-order and second-order logical relations using LAPP method, we define the following quantities for this discretized vector:

(1) Downregulation self-entropy is *H*_−_(*D*) = −∑_*i*=1_^*n*^ *p*_*D*_(*x*_*i*_)log⁡(*p*_*D*_(*x*_*i*_)).
(2) Upregulation self-entropy is *H*_+_(*D*) = −∑_*i*=1_^*n*^ *p*_*D*_(*x* ^*i*^)log⁡(*p*_*D*_(*x* ^*i*^)).

Then self-entropy is *H*(*D*) = *H*_−_(*D*) + *H*_+_(*D*), where *p*_*D*_(*x*_*i*_) and *p*_*D*_(*x* ^*i*^) are the corresponding frequency of components *x*_*i*_ and *x* ^*i*^, respectively, in vector *D*. Considering two vectors *D* = (*d*_*m*1_′,…, *d*_*mp*_′,…, *d*_*mn*_′) and *B* = (*b*_*m*1_′, *b*_*m*2_′,…, *b*_*mn*_′), we define the following joint entropies:

(3) *H*_+_(*D*, *B*) = −∑_*i*=1_^*n*^ *p*_*D*,*B*_(*x* ^*i*^, *x* ^*j*^)log⁡(*p*_*D*,*B*_(*x* ^*i*^, *x* ^*j*^)),
(4) *H*_−_(*D*, *B*) = −∑_*i*=1_^*n*^ *p*_*D*,*B*_(*x*_*i*_, *x*_*j*_)log⁡(*p*_*D*,*B*_(*x*_*i*_, *x*_*j*_)),
(5) *H*_+_^−^(*D*, *B*) = −∑_*i*,*j*=1_^*n*^ *p*_*D*,*B*_(*x* ^*j*^, *x*_*i*_)log⁡(*p*_*D*,*B*_(*x* ^*j*^, *x*_*i*_)),
(6) *H*_−_^+^(*D*, *B*) = −∑_*i*,*j*=1_^*n*^ *p*_*D*,*B*_(*x*_*i*_, *x* ^*j*^)log⁡(*p*_*D*,*B*_(*x*_*i*_, *x* ^*j*^)).

And total joint entropy is *H*(*D*, *B*) = *H*_+_(*D*, *B*) + *H*_−_(*D*, *B*) + *H*_+_^−^(*D*, *B*) + *H*_−_^+^(*D*, *B*), where *p*_*D*,*B*_(*x*_*i*_, *x*_*j*_), *p*_*D*,*B*_(*x* ^*i*^, *x* ^*j*^) are the corresponding frequencies of component *x*_*i*_ *x*_*j*_ in vectors *D* and *B*, respectively. For discretized vectors *A* and *B*, the uncertainty coefficient (*U* value) is defined as

(1) $$\begin{array}{c} U\left(\;B\;|\;A\;\right)=H\left(\;B\;\right)+H\left(\;A\;\right)-\frac{H\left(A,B\right)}{H\left(B\right)}. \end{array}$$

This quantity informs on the probability that *A* regulates *B*. Note that for simplicity *A* and *B* denote not only expression vectors but also the corresponding genes. The first-order logical relationship between genes *A* and *B* is determined as

(2) $$\begin{array}{c} U\left(\;B\;|\;f_{1}\left(\;A\;\right)\;\right)=H\left(\;B\;\right)+H\left(\;f_{1}\left(\;A\;\right)\;\right)-\frac{H\left(B,f_{1}\left(A\right)\right)}{H\left(B\right)}, \end{array}$$

where *f*_1_ is the proper functions of first-order logic of *A* to *B*. The uncertainty coefficient of (*A*, *B*) → *C* is

(3) UC ∣ f2A,B=HC+Hf2A,B−HC,f2A,BHC,

where *f*_2_ is one of the proper functions of second-order logic of *A* to *B*. Table 2 lists ten types of proper functions and the corresponding algebraic operations. Using these operations on *A* and *B*, we get *C*. Table 2 also gives the algebraic representation of three basic operators for multiple values: logical NOT is represented as ¬(*a*_*i*_ = *x*_*k*_  or  *x* ^*k*^) = (*a*_*i*_ = *x* ^*k*^  or  *x*_*k*_), logical AND and OR can be represented as min⁡(*a*_*i*_, *b*_*i*_), max⁡(*a*_*i*_, *b*_*i*_), where *a*_*i*_, *b*_*i*_ are the *i*th component of *A*, *B*.

**Figure 1:**
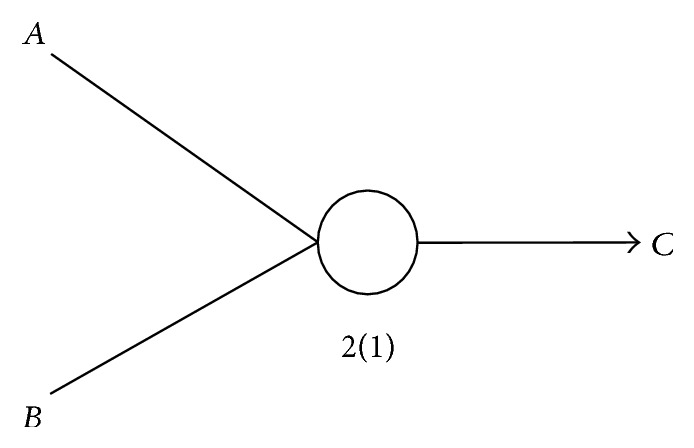
An example of a logic triplet; the circle in the middle represents the logic type.

We normalize the *U* value for each gene triplet and database by replacing it with *U*/max⁡*U*, where max⁡*U* is the maximum value in the own database. For simplicity, the normalized values are also denoted by *U*. The condition *U*(*C*∣*f*_2_(*A*, *B*)) ≥ max{*U*(*C*∣*A*) + *t*, *U*(*C*∣*B*) + *t*} is used to filter out all gene triplets. The combination requires gene *C* to be better predicted from genes *A* and *B* together than just gene *A* alone or gene *B* alone.  Figure 1 shows an example for a gene triplet, for which the logical AND operation on gene *A* and gene *B* is denoted by *A*∨*B* → *C*. As in the LAPP method, all such gene triplets, with the corresponding the *U* values, give rise to the gene logic networks further studied in our present work.

#### 2.2.2. Structural Parameters Definition of Logic Network

In fact, the logic network can be seen as a directed and weighted network without multiple edges and self-loops. Let *G* = (*V*, *E*, *W*) be a logic network with gene node-set *V*, directed edge-set *E*, and function *W* : *E* → *R* that assigns each edge *e* ∈ *E* and weight *W*(*e*) ∈ *R*. In fact, the edge weights can be interpreted as the uncertainty coefficients that express interaction strength between gene triplets. Various structural parameters have used to study network structure in complex network [27]. For the logic network, structural parameters are detected again including average degree, average path length, average clustering coefficient, and average betweenness to capture structural characteristics of the logic network from different angles. 

*(1) Average Degree (D*). The degree of a node is the number of nodes adjacent to it. The average degree is the average value of the degrees of all nodes. For the logic network, the definitions of in-degree, out-degree need to be reconsidered based on the principle that the sum of in-degree and that of out-degree of all the nodes in a network are equivalent. Based on the principle, in-degree and out-degree can be defined according to second-order logical relationship; if there are *k* activations of *C*, then in-degree of *C* increases by *k*/2. However, the out-degrees of *A* and *B* are determined by the proportion of their contributions to the second-order logical relationship. Here we can assume that the proportion contribution to the second-order logical relationship from *A* and that from *B* are always equivalent. In other words, the out-degree increment of *A* is the same as that of *B*.

*(2) Average Clustering Coefficient (C).* For a certain node with second-order logical relationships, we need to define doublets of second-order logical relationships to measure the clustering coefficient. A doublet of second-order logical relationships is a combination of two second-order logical relationships with at least one common node.

**Figure 2:**
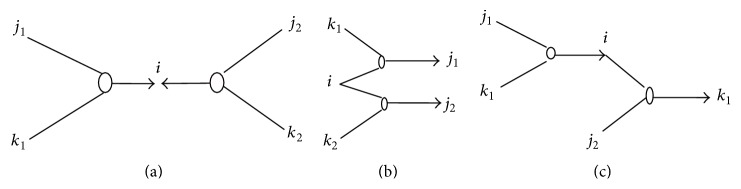
(a) Double-in second-order logic doublets centered on *i*. (b) Double-out second-order logic doublets centered on *i*. (c) In-out second-order logic doublets.

**Figure 3:**
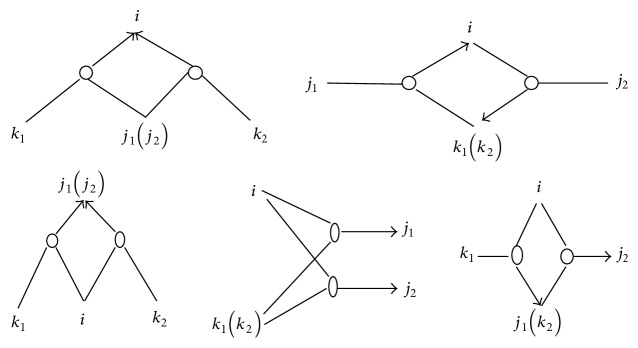
Strong second-order logic doublets centered on *i*.

If the common node is *v*, we call this second-order logic doublet centered on *v*. Figure 2 shows all possible second-order logic doublets centered on *v*. These three types of second-order logic doublets are named according to the different positions of *v* as “both-in,” “both-out,” and “in-out” doublets. If there are two common nodes in a second-order logic doublet, we call the doublet strong connected.  Figure 3 shows all possible strong connected second-order logic doublets centered on *v*. The number of second-order logic doublets (including both-in, both-out, and in-out doublets) centered on *v* is denoted by *σ*_doub_(*v*), and the number of strong connected second-order logic doublets is denoted by *σ*_sc-doub_(*v*). The clustering coefficient of node *v*, denoted by *C*_doub_(*v*), in a logic network with second-order logical relationships is defined as *C*_doub_(*v*) = *σ*_sc-doub_(*v*)/*σ*_doub_(*v*). So the average clustering coefficient of network is defined as $\bar{C_{G}}=\left(1/n\right)\sum_{v\in G}^{}C_{doub}\left(v\right).$

*(3) Average Path Length (L*). Path and its length should be reconsidered according to the different second-order logic types: AND, OR, and XOR. Take a second-order logical relationship (*A*, *B*) → *C*, and an arbitrary node in *V* other than *C* (say *X*), the shortest directed path *p*_*XC*_, and the distance *d*_*XC*_ from *X* to *C* are defined as follows.

**Case 1 (second-order logic type is AND).**

Namely, nodes *A* and *B* regulate node *C* cooperatively.

(1) *X* is *A* or *B* (say *A* without loss of generality). The shortest paths from *X* to *B* are denoted by *p*_*XB*_, if there is at least one directed path from *X* to *B*. The directed path *p*_*XC*_ from *X* to *C* arrives at *B* through *p*_*XB*_ and then the second-order logical relationship to *C*. The distance *d*_*XC*_ from *X* to *C* is the total sum of *d*_*XB*_ and *d*_(*A*, *B*)→*C*_, where *d*_(*A*, *B*)→*C*_ is the length of the second-order logical relationship. In our study, *d*_(*A*, *B*)→*C*_ is estimated by the reciprocal of the uncertainty coefficient of this second-order logical relationship. On the other hand, if *v* and *B* are not connected by a directed path, there is no path from *v* to *C*.
(2) *X* is neither *A* nor *B*. *X* is reachable from at most one of *A* and *B*. Then there is no directed path starting from *X* and ending at *C*. *X* is reachable from both *A* and *B*. Then there is at least one directed path connecting *X* and *C*. The distance *d*_*XC*_ from *X* to *C* is *d*_*XC*_ = max{*d*_*XA*_, *d*_*XB*_} + *d*_(*A*, *B*)→*C*_. The directed path *p*_*XC*_ first reaches the nearer one of *A* and *B* followed by the other and finally *C*.

**Case 2 (second-order logic type is OR).**

Either *A* or *B* can regulate *C* independently. The distance *d*_*AC*_ from *A* to *C* is estimated by the probability that activation of *C* results from *A*. The next step is to distribute the uncertainty coefficient *U*(*C*∣*f*_2_(*A*, *B*)) of the second-order logical relationship. Calculate the uncertainty coefficients caused by *A* and *B*, denoted by *U*(*C*∣*A*) and *U*(*C*∣*B*), respectively, as follows:

(4) UC ∣ A=12·UC ∣ f2A,B,UCB=12·UC ∣ f2A,B.

On the basis of above obtained uncertainty coefficients, the shortest directed path *p*_*XC*_ and the distance *d*_*XC*_ from *X* to *C* can be determined as follows.

(1) *X* is *A* or *B* (say *A* without loss of generality). There is at least one path starting with *X* and ending at *B*. Choose one of the shortest directed paths from *X* to *B* randomly, denoted by *p*_*XB*_. The total sum of *d*_*XB*_ and the reciprocal of *U*(*C*∣*B*) are denoted by *d*_*XC*_^(1)^ and *d*_*XC*_^(2)^, respectively. If *d*_*XC*_^(1)^ is less than *d*_*XC*_^(2)^, the shortest directed path *p*_*XC*_ goes directly from *X* to *C* through the second-order logical relationship, and the distance *d*_*XC*_ equals *d*_*XC*_^(1)^. Otherwise, *p*_*XC*_ first arrives at *B* and then *C*, and *d*_*XC*_ is equal to *d*_*XC*_^(2)^. There is no path from *A* to *B*. The path from *X* to *C* is only the second-order logical relationship, and the distance *d*_*XC*_ equals the reciprocal of *U*(*C*∣*A*).
(2) *X* is neither *A* nor *B*. There is at least one path starting from *v* towards *A* or *B*. Therefore, the distance from *X* to *C* is *d*_*XC*_ = min{*d*_*XA*_ + *d*_(*A*, *B*)→*C*_, *d*_*XB*_ + *d*_(*A*, *B*)→*C*_}. The shortest directed path from *X* to *C* is the corresponding path to the choice of distance. Note that when *X* is unreachable from *A* (or *B*), *d*_*XA*_ (or *d*_*XB*_) is infinite. Neither *A* nor *B* is reachable from *X*. The distance from *X* to *C* is infinite and no path connects them in this case.

**Case 3 (second-order logic type is XOR).**

Both *A* and *B* can activate *C* cooperatively or independently. Therefore, XOR type of second-order logical relationship is a combination of AND type and OR type. However, when only one of *A* and *B* is reachable from *X*, the condition is the same as OR logic. When both *A* and *B* are reachable from *X*, or *X* is *A* or *B*, it is the same as AND logic.

According to the definition above, all shortest directed paths and all distances can be found in a logic network. And the average path length of a logic network is defined as $\bar{L_{G}}=\left(1/\left|D\right|\right)\sum_{\left(s,t\right)\in D}^{}d_{st}$, where *D* is the set of ordered pairs of nodes and the distance from the first one to the second one is finite; that is, *D* = {(*s*, *t*)∣*s*, *t* ∈ *V*, *d*_*st*_ < +*∞*}.  |*D*| is the number of the elements in *D*.

*(4) Average Betweenness (B*). Betweenness centrality [28] is one indicator of a node's centrality in complex network. It is equal to the number of shortest paths from all vertices to all others that pass through that node. A node with high betweenness centrality has a large influence on the transfer of items through the network, under the assumption that item transfer follows the shortest paths. Let *L*_*G*_ be the set of all the shortest paths (allowing more than one shortest paths between two nodes) in the logic network *G*. If node *v* appears at least once in a directed path *p*_*st*_ starting with *s* and ending at *t*, then *v* is referred to as intermediate node in this path, denoted by *v* ∈ *p*_*st*_. The standard betweenness of *v*, denoted by *C*_Logic_(*v*), is defined as

(5) $$\begin{array}{c} C_{Logic}\left(\;v\;\right)=\frac{\sum_{\begin{array}{c} s\in V,s\neq v,\\ t\in V,t\neq v \end{array}}^{}\left|\left\{p_{st}\;|\;v\in p_{st}\right\}\right|}{\left|L_{G}\right|}. \end{array}$$

The standard betweenness centrality ranges from zero to one. Higher betweenness means larger possibility to appear in the shortest paths. It is more important in the structure and information transfer of a network. Therefore betweenness can help to discover crucial nodes that may have significant impacts on the structural characteristics of the logic network.

#### 2.2.3. Community Structures in the Logic Network

Another important feature of complex network is its community structures, which depict the organization of vertices into clusters, with many edges joining vertices of the same cluster and comparatively few edges joining vertices of different clusters. The community is a good tool to describe network structures and provides better understanding of network functions. Several researchers [29–31] proposed algorithms to detect community structures. Newman fast algorithm [32] is a greedy modularity algorithm starting from a set of isolated nodes. The links of the original graph are iteratively added such that the largest possible increase of the modularity at each step is achieved. The fast algorithm is used to find the community structure in the logic network. In addition, the inside-to-outside-of-community ratio *α* = *n*_1_/*n*_2_ is used to evaluate how close of community connections in the network, where *n*_1_ is number of edges within a community and *n*_2_ is the number of edges cooperating the internal and external node in a community.

## 3. Results

**Figure 4:**
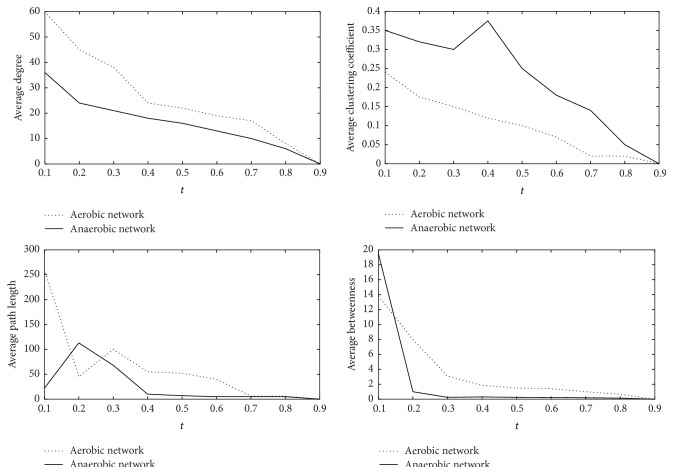
The curves of four parameters with the change of threshold *t* from 0.1 to 0.9 between the aerobic network and the anaerobic network.

In order to highlight the characteristics of the logic network structures, we contrast the change curves (Figure 4) of four structural parameters along with threshold *t* from 0.1 to 0.9 between the aerobic network and the anaerobic network with step length 0.1. We find that the average degree, average clustering coefficient, and average betweenness of the aerobic network are greater than those of the anaerobic network for all thresholds. It can be seen that the average path length of the aerobic network is greater than that of the anaerobic network in some threshold ranging from 0.3 to 0.7. The significant changes of parameters mean that the energy metabolism conditions for* Saccharomyces cerevisiae* in aerobic and anaerobic respirations actually differ on the molecular level.

Each node of the logic network corresponds to a different structural parameter. From this, we obtain the degree, clustering coefficient, betweenness, and path length of each node. By calculating and ranking the difference values of the four parameters for each node, we capture the top five gene nodes. In Table 3, *D*-Difference, *L*-Difference, *C*-Difference, and *B*-Difference denote the difference values of the four structural parameters, respectively. Finally we get the intersection of these gene nodes which includes genes ATP6, YIG1, RGI2, BAG7, and COX1. The structural parameters of these genes change significantly comparing the aerobic network with the anaerobic network. For example, some genes are allocated a higher degree in the aerobic network than in the anaerobic network. That is to say, these genes have great contribution to degree structure changes for the two networks, so we define them as the structural key genes.

If gene *A* connects other genes by a certain second-order logic relationship, then these genes together from one set are denoted by *S*_*A*_. For example, if *C*∩*D* → *A*, then *C* ∈ *S*_*A*_, *D* ∈ *S*_*A*_; if *A*∩*F* → *G*, then *G* ∈ *S*_*A*_. If the gene of *S*_*A*_ has a particular function, then we predict that gene *A* also has the same function. David Gene Functional Classification analysis is carried out on *S*_*A*_. The second column of Table 4 shows that the functional annotation of these genes has been detected in David database while the final column lists the predicted functions of them.

Table 5 shows nonisolated nodes, the numbers, and modularity of community structures. Utilizing Newman fast algorithm, we find that the aerobic network has two obvious community structures. The modularity is 0.3756 including 47 and 15 nonisolated nodes. The corresponding ratio of inside and outside of community is 87.3 and 19.8, respectively. There are three community structures in anaerobic network, including 13, 16, and 5 nodes. The corresponding ratio of inside and outside of community is 1.8, 6.0 and 0.57 (see Table 6).

## 4. Conclusions

By David Gene Functional Classification, we predict that the genes ATP6 COX1 BAG7 and YIG1 are involved in the yeast mitochondrial respiratory chain while RGI2 is involved in membrane transportation (Table 4). In fact, ATP6 [33] belongs to the family of genes, which is called the mitochondrial respiratory chain complex. It participates in the respiratory chain and provides information for synthesis of a protein encoded by mitochondrial DNA that is essential for normal mitochondrial function. YIG1 [34] encodes protein and is involved in regulating anaerobic glycerol metabolism in* Saccharomyces cerevisiae*. Deletion or overexpression of YIG1 significantly affects growth yield or glycerol yield in anaerobic batch cultures. This is consistent with the previously proposed low flux control exerted at the Gpp level. BAG7 [35] is involved in the energy metabolism under aerobic conditions; its expression is induced under carbon limitation and suppressed under high glucose; COX1 [36] belongs to the family of cytochrome C-oxidase, catalyzing the reduction of oxygen to water in the respiratory chain. RGI2 [37] is a protein of unknown function associated with metabolism under conditions of aerobic respiration; its expression is induced under carbon limitation and suppressed under high glucose. It is involved in the control of metabolism and significantly contributes to cell fitness, especially under respiratory growth conditions.

**Figure 5:**
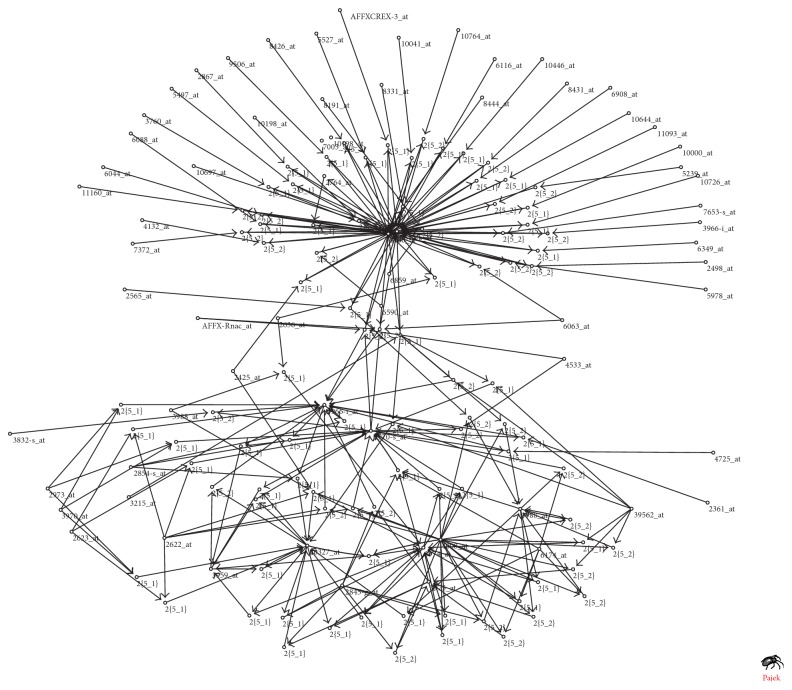
Spider diagram of the aerobic network.

**Figure 6:**
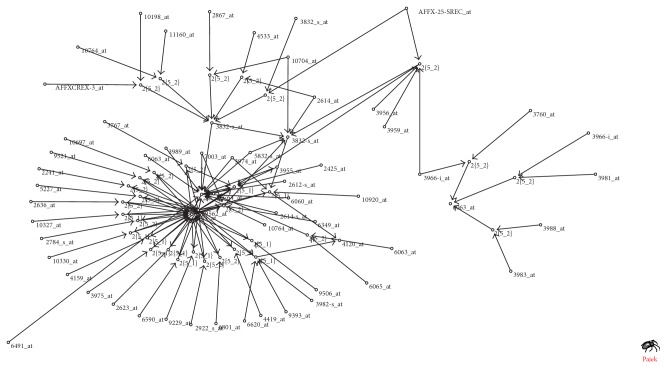
Spider diagram of the anaerobic network.

Some biological functions of organism are realized by gene interactions of proteins. These genes are closely linked, thus revealing the community phenomenon of the logic network. We produce the spider diagrams in Figures 5 and 6 using the Pajek software (http://mrvar.fdv.uni-lj.si/pajek/). The aerobic network is shown to have two distinct communities while the anaerobic network has an obvious community. These communities may correspond to certain biological functions. So we apply the David Gene Functional Classification analysis to these communities. The result reveals that, in the first community of the aerobic network, seven genes including 4533_at, 5527_at, 8444_at, 8426_at, 10446_s_at, 6116_at, and 6908_at are related to the cell cycle function. Ten genes including 2565_s_at, 2498_s_at, 3215_f_at, 8822_at, 6044_at, 2361_s_at, 6590_at, 10644_at, 10041_at, 8431_at, and 2425_at are associated with the DNA transcription. In the second community of the aerobic network, 12 genes including 3975_at, 3970_s_at, 3974_at, 3959_at, 4008_at, 3988_at, 3987_at, 2623_s_at, 2622_s_at, 2845_g_at, 2793_s_at, and 3966_i_at are involved in the mitochondrial respiratory chain.

## 5. Discussions

For the nonbinary gene expression profiles, this work gives a novel approach in calculating the self-entropy (including downward self-entropy, upregulation self-entropy) and joint entropy for gene vectors. LAPP method is utilized to find all gene triplets. Furthermore the gene logic networks are constructed. The relationships between the structure and the function of the network could help us to understand metabolisms of* Saccharomyces cerevisiae* on the molecular level. To analyze the structural difference between two networks, parameters such as average degree, average clustering coefficient, average path length, and average betweenness, which reveal that the second-order logical types have significant differences among the different experimental gene sets, have been generalized to the networks. The differences may provide us with a new idea and some reference to the biologists on their research work. However, how the other analytical methods, such as community structures, can be generalized into the logic networks is still an interesting issue. By applying the Newman fast algorithm to the logic networks, we find that the aerobic network has two significant communities, while the anaerobic network has only one significant community. Furthermore the David Gene Functional Classification shows that one of two communities possibly relates to cell cycle and cell replication functional group; the other possibly correlates mitochondrial respiratory chain functional group in the aerobic network.

## Figures and Tables

**Table 1:** The data source.

Group	Platform	Database	Sample size
Aerobic	GPL90	GSE11452	42
Anaerobic	GPL90	GSE11452	52

**Table 2:** Illustration of 2-order logical types.

Type	Illustration	The proper function	Representation
1	*C* is present iff *A* and *B* are both present	*C* = *A*∧*B*	min(*a*_*i*_, *b*_*i*_)

2	*C* is present iff *A* is absent or *B* is absent	*C* = ¬(*A*∧*B*)	max(¬*a*_*i*_, ¬*b*_*i*_)

3	*C* is present iff *A* is present or *B* is present	*C* = (*A*∨*B*)	max(*a*_*i*_, *b*_*i*_)

4	*C* is present iff *A* is absent and *B* is absent	*C* = ¬(*A*∨*B*)	min(¬*a*_*i*_, ¬*b*_*i*_)

5	*C* is present iff *A*(*B*) is absent and *B*(*A*) is present	*C* = (¬*A*∧*B*), *C* = (*A*∧¬*B*)	min(¬*a*_*i*_, *b*_*i*_), min(*a*_*i*_, ¬*b*_*i*_)

6	*C* is present iff *A*(*B*) is absent or *B*(*A*) is present	*C* = (¬*A*∨*B*), *C* = (*A*∨¬*B*)	max(¬*a*_*i*_, *b*_*i*_), max(*a*_*i*_, ¬*b*_*i*_)

7	*C* is present iff *A* and *B* are both present or *A* and *B* are both absent	*C* = (*A*⟷*B*)	max[max(¬*a*_*i*_, *b*_*i*_), max(*a*_*i*_, ¬*b*_*i*_)]

8	*C* is present iff one of either *A* or *B* is present	*C* = ¬(*A*⟷*B*)	max[min(¬*a*_*i*_, *b*_*i*_), min(*a*_*i*_, ¬*b*_*i*_)]

**Table 3:** Difference of parameters for some genes between the aerobic and the anaerobic network.

Gene	COX1	ATP6	COB	BAG7	YIG1	RGI2

*D*-Difference	22	22	21	20	16	15

Gene	COX1	YIG1	BAG7	BI3	ATP6	RGI2

*L*-Difference	12	11	11	10	9	7

Gene	COB	ATP6	COX1	BAG7	YIG1	RGI2

*C*-Difference	2.320	2.194	2.183	2.110	2.065	1.910

Gene	BAG7	YIG1	RGI2	COX3	COX1	ATP6

*B*-Difference	0.286	0.263	0.255	0.207	0.186	0.172

**Table 4:** Biological function of structural key genes.

Gene	Biological function annotation	Predicted function
ATP6	Mitochondrial membrane ATP synthetase, participating in the respiratory chain	Involved in yeast mitochondrial respiratory chain

RGI2	Associated with energy metabolism under condition of aerobic respiration	Involved in membrane transport

COX1	Cytochrome C-oxidase, catalyzing the reduction of oxygen to water in the respiratory chain	Involved in yeast mitochondrial respiratory chain

BAG7	Signal conduction function, activation of RHO1 which can regulate Gsc2p and Fks1p	Involved in yeast mitochondrial respiratory chain

YIG1	Compiling protein lipid interactions under anaerobic conditions and associated with the production of glycerol metabolism	Involved in yeast mitochondrial respiratory chain

**Table 5:** Modularity of the logic networks.

Group	Nonisolated nodes	Number	Modularity
Aerobic network	67	2	0.3756
Anaerobic network	44	3	0.2842

**Table 6:** Communities of the logic networks.

Group	Community	Nodes in community	Ratio of inside and outside ofcommunity
Aerobic network	Community 1	50	89.3
	Community 2	17	18.8

Anaerobic network	Community 1	11	1.80
	Community 2	28	6.43
	Community 3	5	0.57
